# Neuroprotective effects of methanolic extract from Chuanxiong Rhizoma in mice with middle cerebral artery occlusion-induced ischemic stroke: suppression of astrocyte- and microglia-related inflammatory response

**DOI:** 10.1186/s12906-024-04454-w

**Published:** 2024-04-04

**Authors:** Chiyeon Lim, Sehyun Lim, So-Jung Moon, Suin Cho

**Affiliations:** 1 College of Medicine, Dongguk University, Goyang, 10326 Republic of Korea; 2 Department of Radiology, Massachusetts General Hospital, Harvard Medical School, Charlestown, MA 02129 USA; 3 School of Public Health, Far East University, Eumseong, 27601 Republic of Korea; 4 College of Science & Industry Convergence, Ewha Womans University, Seoul, 03760 Republic of Korea; 5 School of Korean Medicine, Pusan National University, Yangsan, 50612 Republic of Korea; 6 Department of Korean Medicine, School of Korean Medicine, Yangsan Campus of Pusan National University, Yangsan, 50612 Republic of Korea

**Keywords:** Cerebrovascular disease, Ischemic stroke, Cerebral infarction, Herbal medicine, Neuroprotection

## Abstract

**Background:**

In traditional Asian medicine, dried rhizomes of *Ligusticum chuanxiong* Hort. (Chuanxiong Rhizoma [CR]) have long been used to treat pain disorders that affect the head and face such as headaches. Furthermore, they have been used primarily for blood circulation improvement or as an analgesic and anti-inflammatory medicine. This study aimed to investigate the neuroprotective effects of a methanol extract of CR (CRex) on ischemic stroke in mice caused by middle cerebral artery occlusion (MCAO).

**Methods:**

C57BL/6 mice were given a 1.5-h transient MCAO (MCAO control and CRex groups); CRex was administered in the mice of the CRex group at 1,000–3,000 mg/kg either once (single dose) or twice (twice dose) before MCAO. The mechanism behind the neuroprotective effects of CRex was examined using the following techniques: brain infarction volume, edema, neurological deficit, novel object recognition test (NORT), forepaw grip strength, and immuno-fluorescence staining.

**Results:**

Pretreating the mice with CRex once at 1,000 or 3,000 mg/kg and twice at 1,000 mg/kg 1 h before MCAO, brought about a significantly decrease in the infarction volumes. Furthermore, pretreating mice with CRex once at 3,000 mg/kg 1 h before MCAO significantly suppressed the reduction of forepaw grip strength of MCAO-induced mice. In the MCAO-induced group, preadministration of CRex inhibited the reduction in the discrimination ratio brought on by MCAO in a similar manner. CRex exhibited these effects by suppressing the activation of astrocytes and microglia, which regulated the inflammatory response.

**Conclusions:**

This study proposes a novel development for the treatment of ischemic stroke and provides evidence favoring the use of L. chuanxiong rhizomes against ischemic stroke.

**Supplementary Information:**

The online version contains supplementary material available at 10.1186/s12906-024-04454-w.

## Introduction

Cerebrovascular diseases (CVDs) are major causes of death worldwide; acute ischemic stroke is a leading cause of morbidity and mortality in modern society [1–3]. Stroke can be largely classified into cerebral infarction and cerebral hemorrhage. Of these, cerebral infarction is the most common disease, accounting for approximately 80% of all strokes. When ischemic stroke occurs, cerebral inflammation and cell death are induced in the affected region, and the deterioration of mitochondrial functions is activated by harmful stimuli such as brain arterial ischemia and reperfusion [4, 5].

The treatment of stroke is dependent on several factors; the type of stroke is an essential factor in determining the treatment. With ischemic stroke, restoring blood circulation in affected brain areas is a priority and usually requires medications called thrombolytics. Although thrombolytic agents have been used to treat stroke with some success, side effects such as hemorrhage and disadvantages such as narrow therapeutic time window have limited their use. Thus, active research is ongoing on the prevention and treatment of ischemic stroke [4, 6, 7].

In Chinese and Korean traditional medicine, the cause of CVDs, such as cerebral hemorrhage or cerebral infarction, is defined as *qi* stagnation and blood stasis. The medicinal herbs frequently used to treat these conditions include Angelicae Sinensis Radix, Angelicae Gigantis Radix, Chuanxiong Rhizoma (CR), Salviae Miltiorrhizae Radix, Carthami Flos, and Paeoniae Radix [8, 9]. This study aimed to confirm the effectiveness of CR in animal models of cerebral infarction.

CR is the dried rhizome of *Ligusticum chuanxiong* Hort. It belongs to the *Umbelliferae* family. Because the rhizome of this plant was initially described in *Sheng-Nong’s Classic of Materia Medica*, it is considered “*qi* medicine in the blood” in traditional Asian medicine due to its excellent efficacy in stimulating blood circulation and warding off winds. Therefore, CR is a major cardiovascular protective herbal medicine that is used clinically. Furthermore, it is a popular pharmaceutical prescribed for the treatment of atherosclerosis, ischemic stroke, angina pectoris, and hypertension. It also exhibits antioxidant, neuroprotective, and anti-inflammatory activities [8–10].

CR is one of the most frequently used medicinal herbs among prescriptions in traditional Asian medicine to treat patients with CVDs. Moreover, CR affects ischemic brain damage by improving the blood flow of microvessels supplied to the brain; clinical studies are being conducted to evaluate its effects on the recovery of patients from cerebrovascular accidents [11–15].

The middle cerebral artery occlusion (MCAO) model is commonly used to induce a focal cerebral ischemic insult (stroke) in rodents. The MCAO model has relatively high reproducibility among various cerebral infarction models [16, 17]. In this study, the MCAO model was established by inducing cerebral infarction in mice to determine whether the CR suppresses ischemic cerebral infarction. Provided that herbal medicines have several ingredients, it is challenging to specify the activity of an ingredient. Furthermore, because it cannot be specified whether one component exhibits only one activity, we confirmed the evidence that CR can be effectively used for CVDs through network pharmacology analysis and ameliorate MCAO-induced ischemic stroke in experimental mice. Next, we confirmed whether the brain damage could be suppressed by administration of the CR methanol extract (CRex). To confirm the level of pathological changes caused by MCAO and the inhibition of pathological changes by CRex administration, infarct volume, edema, neurological deficit score (NDS), and behavioral changes were observed; changes in neuroinflammation related to astrocyte and microglia localization were confirmed with immunofluorescence staining.

## Materials and methods

### Network pharmacology analysis

Network pharmacology research is commonly conducted based on the traditional Asian medicine databases, represented by the Traditional Chinese Medicine Systems Pharmacology Database and Analysis Platform (TCMSP) [18], which is mainly used in studies related to Chinese medicine that have employed various network pharmacology research methods. In this study, the analysis was performed using the TCMSP database. Expected active components were selected. Next, the absorption, distribution, metabolism, and excretion (ADME) of each component was determined. Finally, pharmacokinetically active compounds with potential potency were selected. Oral bioavailability (OB), which indicates the ability of an orally administered drug to be delivered to the body; drug-likeness (DL), which helps optimize pharmacokinetic properties as an estimate of the number of many potential compounds present; and Caco-2, which evaluates drug absorption in the gastrointestinal tract, are included in ADME variables. Each selected component showed the minimum values of OB ≧ 30%, DL ≧ 0.18, and Caco-2 ≧ 0 that have been recommended by the TCMSP variables [18]. The compound–disease network was generated using Cytoscape (ver 3.10.0), an open software platform for visualizing complex networks.

### Reagents

Phosphate-buffered saline (PBS) was purchased from Bio Basic Inc. (Markham, Ontario, Canada). 2,3,5-triphenyl-tetrazolium chloride (TTC) was obtained from Sigma-Aldrich Co. (St. Louis, MO, USA). The optimal cutting temperature compound cryostat embedding medium was obtained from Thermo Fisher Scientific (Waltham, MA, USA). Primary antibodies against neuronal nuclear (NeuN), glial fibrillary acidic protein (GFAP), ionized calcium-binding adaptor molecule 1 (Iba1) were used (Cat. nos. 94,403, 12,389, and 17,198, respectively; Cell Signaling Technology, MA, USA). Goat anti–mouse IgG H&L (Alexa Fluor 488) and goat anti–rabbit IgG H&L (Alexa Fluor 555) (ab150113 and ab150078, respectively) were purchased from Abcam (Cambridge, UK) for secondary immunofluorescent (IF) detection. The bovine serum albumin (BSA) standard was purchased from Thermo Fisher Scientific.

### Preparation of CRex and oral gavage to mice

The CR used in this study was purchased from Kwangmyeongdang Pharmaceutical Co., Ltd. (Ulsan, Korea). Furthermore, the quality of the purchased CR was confirmed as per the quality standards of the Ministry of Food and Drug Safety of Korea. Next, the CR voucher sample (No. 22CR-129) was refrigerated in the crude drug storage room of the Pusan National University School of Korean Medicine. The raw and dry medicinal CR that weighed 1,034 g, was immersed in crushed 100% methanol for 2 days. The supernatant was filtered using qualitative filter paper (Whatman, No. 2, Tokyo, Japan), and the filtrate was lyophilized using a rotary evaporator and freeze dryer. Then, 77.5 g (7.5% yield) of lyophilized CRex was stored at − 20 °C. A CRex voucher specimen (No. 2022Ex-CR-02) has been deposited in the Plant and Extract Bank of Pusan National University School of Korean Medicine for future reference.

Mice were allocated (at least five mice per group) to a sham-operated group; a transient MCAO-operated, but not CRex-treated group (the MCAO control group); and MCAO-operated and CRex-treated groups, which were pretreated with CRex at 1,000 or 3,000 mg/kg at 1 h (single administration) prior to MCAO or with CRex at 300, 1,000 or 3,000 mg/kg at 1 h and 24 h (two administrations) prior to MCAO, respectively. To administer the above three doses to mice, CRex was dissolved in distilled water to concentrations of 30, 100, or 300 mg/mL, respectively, and administered in proportion to the body weight of the mouse. For instance, for mice weighing 20 g, a dose of 0.20 mL was administered, while for mice weighing 25 g, a dose of 0.25 mL was given. The administration dosage was adjusted proportionally based on the body weight for each concentration. The mice in the sham-operated and MCAO groups received the same amount of physiological saline as that administered with CRex in the CRex-treated group.

### Experimental animals and MCAO induction

**Fig. 1:**
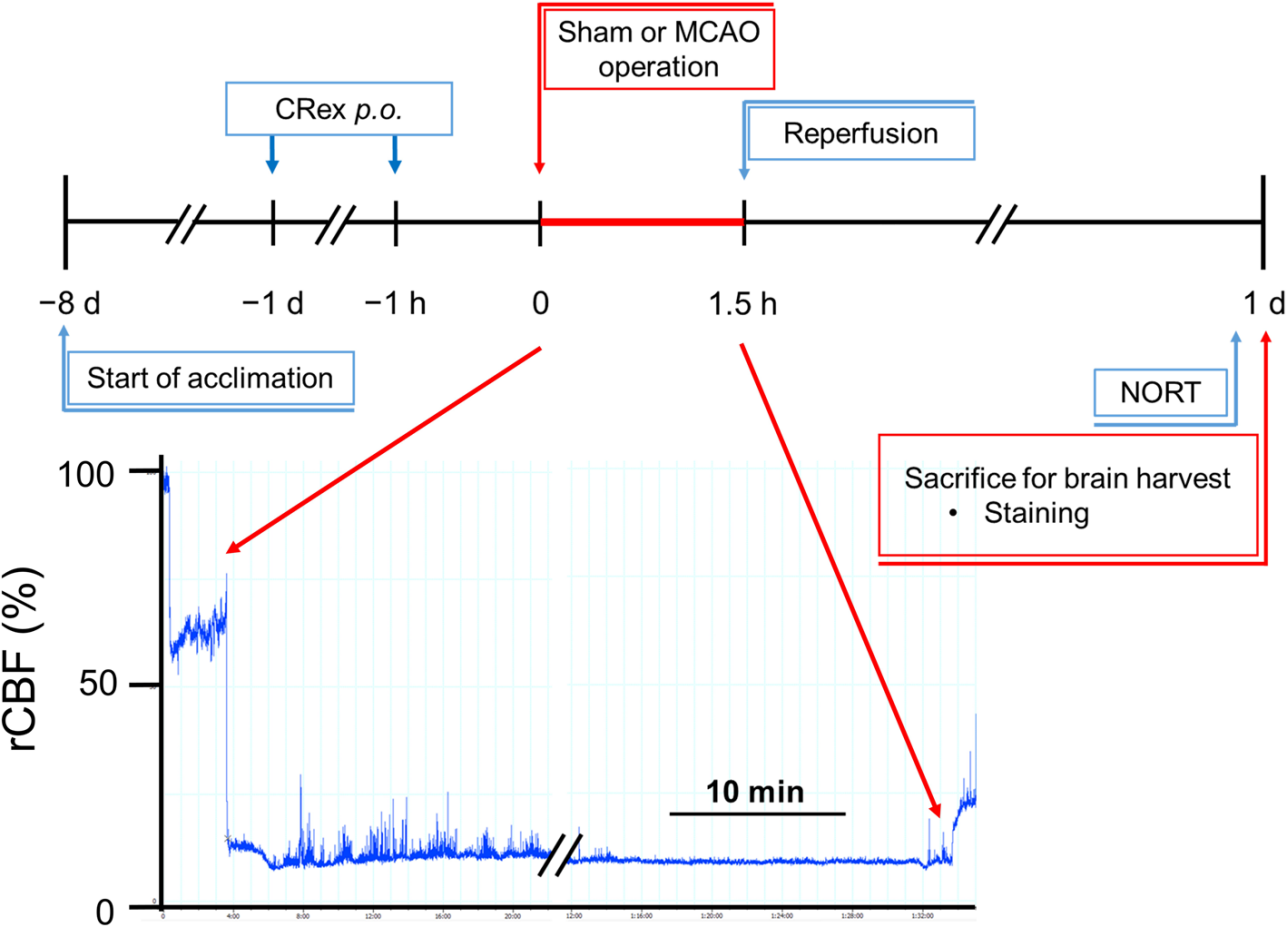
Experimental scheme of the 1.5-h middle cerebral artery occlusion model. The mice were acclimated for 1 week in our animal facility before study commencement. Pretreatment study was performed to determine the neuroprotective effect of orally administered methanolic extract of Chuanxiong Rhizoma (CRex). NORT, novel object recognition test

Fifty specific-pathogen-free 8-week-old C57BL/6 male mice (Samtako Bio, Osan, Korea), weighing 20–22 g were used during this study. This study adheres to ARRIVE guidelines (https://arriveguidelines.org) for the care and use of the animals. The Ethics Committee for Animal Care and Use at the Pusan National University approved all the procedures used in this study (Approval No. PNU 2018–2113). Furthermore, all procedures were certified by the Korean Association of Laboratory Animal Care. The MCAO protocol and materials used were in accordance with previous reports [19, 20]. Intraluminal monofilaments were inserted into the origin of MCA through the left carotid artery of experimental mice and during the operation, a laser Doppler blood flow system (moorVMS-LDF; Moor Instruments, Devon, UK) was used to monitor the relative cerebral blood flow (rCBF). An rCBF reduction of ≧ 80% for 1.5 h was considered to represent successful MCAO induction (Fig. 1). Overall, 63 mice underwent MCAO, 14 mice underwent sham surgery, and 1 mouse died within 24 h of MCAO surgery. Different researchers independently conducted surgeries on experimental mice and performed several assays and statistical analyses. Finally, these researchers, who participated in each step, analyzed the results of the study together.

### Measurement of body weight and physiological parameters

The mice were weighed before and 24 h after MCAO surgery. Following behavioral measurements, blood samples were collected under deep anesthesia via cardiac puncture and were centrifuged at 1,500 × *g* for 15 min at 4 °C, to obtain plasma. Measurement of the plasma concentrations of electrolytes, such as sodium (Na^+^), potassium (K^+^), and chloride (Cl^−^), using an electrolyte analyzer (Dri-Chem 3500i, Fuji, Japan) enabled monitoring of potential electrolyte imbalances.

### Measurement of MCAO-induced cerebral infarct and edema area

Furthermore, 24 h after starting MCAO, the experimental mice were euthanized using CO_2_; Brains of the mice were excised on ice to keep the tissue fresh. Next, the excised brains were sectioned at 1 mm thickness using a mouse brain matrix (Kent Scientific, CT, USA), stained with a solution of 2% 2,3,5-triphenyltetrazolium chloride (TTC), fixed in 10% formalin for at least 2 h, and photographed using a digital camera. TTC stains viable tissue red, whereas the necrotic areas remain white. The cerebral infarct and edema area in each section were analyzed and quantified via the image analysis software (Digimizer, Ostend, Belgium). The increase in area resulting from cerebral injury was measured for cerebral edema and cerebral infarction and expressed as the rate of infarct area occurring in the ipsilateral region [20–22].

As we had demonstrated previously [20], the unstained regions of the fixed brain slices were deemed to be infarcted, and the infarct volume was represented as a percentage of the normal area of the unaffected contralateral hemisphere. The overall size of the infarction was determined by numerically integrating the area of marked pallor, which was measured in 10 consecutive 1-mm coronal sections. The tissue swelling factor was accounted using the following formula: corrected infarct size = infarct size × contralateral hemisphere size / ipsilateral hemisphere size. The degree of left cerebral hemisphere edema was assessed after following MCAO for 24 h. The volumes of both hemispheres were computed in arbitrary units (pixels) from the summation of coronal slice areas based on a digital camera image (Canon EOS M5, Canon Inc. Tokyo, Japan). Brain edema was quantified as the volume of the affected area of the ipsilateral hemisphere.

### NDS and forepaw grip strength test

NDS were determined as described in our previous study [20]. Briefly, the following 5-point scale: 0, no neurological deficit; 1, incomplete extension of the right forepaw; 2, no problem with voluntary movements, but turning to the right when the tail is pulled; 3, walking or circling to the right side and hypersensitivity to mechanical stimulation to the tail; and 4, no response to mechanical stimulation or stroke-related death occurs, was adopted in this study. Following the completion of the NDS test, the mice were placed on a wire grid attached to a custom-made grip test device. Both front paws of the mice grabbed the grid. Then, the grip was slowly pulled until released. The maximum force generated was recorded in grams. All tests were performed on each mouse in triplicates, and the average value was regarded as the measured value.

### Behavioral disorder assessment using the novel object recognition test (NORT)

NORT is a test which helps to assess a rodent’s preference for novel objects over familiar objects [23, 24]. Each mouse was allowed to explore the object-free open-field arena (40 × 40 × 40 cm (height) gray box) for 10 min (Supplementary Fig. 1, Fig. S1) during the adaptation phase. In the first trial, two identical objects were placed in the two opposite corners (zones 2 and 3 in Fig. S1) of the test arena and the experimental mice were allowed to explore the two objects for 20 min. Following 30 min, a second trial was conducted, wherein the mice were placed in the arena again, and one of the identical objects (familiar [F]) provided in the first trial was replaced with a novel (N) object. The behavior of each mouse during the 20-min period spent exploring each object was recorded. The analyses of object search time and discrimination ratio (DR) were performed for each experimental group using the following formula: Total N h / (N h + F h) × 100. The two identical objects and arenas were washed using 70% ethanol between trials.

### Cardiac perfusion and brain cryosection

The mice were euthanized via CO_2_ inhalation, and cardiac perfusion/fixation was conducted using PBS containing 4% paraformaldehyde (PFA). The brain samples were fixed, excised, soaked in 10% PFA, containing 10–30% sucrose, for 3 days at 4 °C, and cryosectioned at 25 μm with a cryostat (CM3050S, Leica, Wetzlar, Germany).

### IF staining of brain sections

The sections of the brain were dried on a slide warmer, incubated with a blocking buffer (5% BSA) for 1 h at 25 °C, and washed using a blocking buffer. Then, the sections were incubated with diluted primary antibodies against NeuN, GFAP, and Iba1 (1:600, 1:400, and 1:200, respectively) overnight at 4 °C. The primary antibodies were washed three times with PBS for 5 min, and the diluted secondary antibodies were dropped on the sections at 25 °C for 2 h. The secondary antibodies were washed three times with PBS for 5 min each. After dropping the mounting medium with DAPI (ab104139, Abcam), the sections were covered using cover slides. The edges of the coverslips were sealed with nail polish. The samples were observed under a fluorescence microscope (Ni-U, Nikon, Tokyo, Japan) and then stored in a refrigerator at 4 °C for long-term storage. Marker expression was calculated using the positive cell count of immuno-intensity per unit area (1 mm^2^) of the brain. Protein expression was determined by measuring the fluorescence intensity following antibody staining using Digimizer version 4.6.1 (MedCalc Software Ltd, Ostend, Belgium).

### Statistical analysis

One-way analysis of variance was used to evaluate whether the differences in mean values among the groups of mice were significant or not using Sigmaplot (version 12.0; Systat Software Inc., CA, USA). The Holm–Sidak test was used for the post hoc analysis of non-normally distributed data. Results are presented as means ± standard deviation (SD), and differences were considered significant at *p* < 0.05.

## Results

### Network pharmacology analysis for compound-target disease network of CR

**Fig. 2:**
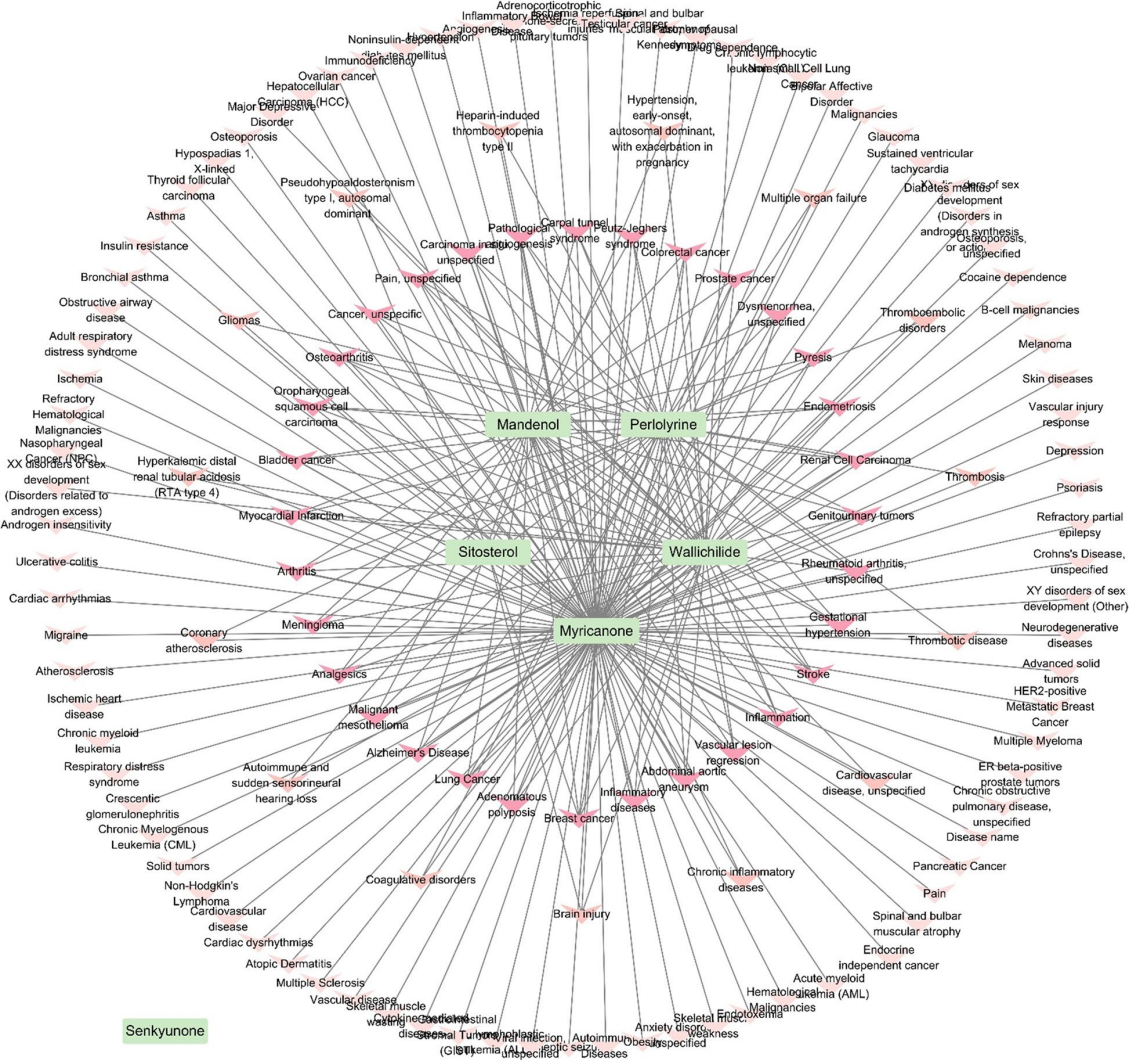
Compound–disease networks of areas (CR). The active CR compounds are represented by the light-green nodes (rectangle-shaped nodes), whereas diseases are represented by the red nodes (V-shaped nodes). The interactions between each compound and diseases it targets are depicted by the gray-colored lines

In total, 189 compounds CR-related compounds were identified in the TCMSP. Following the selection of components using the ADME variables, six compounds were extracted; however, among them, senkyunone was identified as having no target disease (Table 1; Fig. 2). In total, 133 target diseases were connected to the five components of CR, and among them, myricanone had 125, wallichilide had 41, perlolyrine had 40, mandenol had 34, and sitosterol had 6 target diseases. The target proteins and diseases on which each component is expected to act are schematized in the form of an Euler diagram for easy identification of the overlapping aspects (Fig. S2). Moreover, results revealed that breast cancer, inflammatory diseases, and vascular legion regression were ranked high among diseases in the large compound-target disease network. Brain diseases such as stroke and Alzheimer’s disease are related to each of the four compounds, and diseases related to brain injury and thrombosis are also related to relatively different compounds among the 133 diseases, which are predicted to be influenced by CR (Fig. 2).

**Table 1:** Active compounds of Chuanxiong Rhizoma, the dried rhizome of *Ligusticum Chuanxiong*

Mol ID	Molecule name	MW	OB (%)	Caco-2	DL	Structure
MOL001494	Mandenol	308.56	42	1.46	0.19	
MOL002135	Myricanone	356.45	40.6	0.67	0.51	
MOL002140	Perlolyrine	264.3	65.95	0.88	0.27	
MOL002151	Senkyunone	326.52	47.66	1.15	0.24	
MOL002157	Wallichilide	412.57	42.31	0.82	0.71	
MOL000359	Sitosterol	414.79	36.91	1.32	0.75	

Abbreviations: *Mol* Molecule, *MW* Molecular weight, *OB* Oral bioavailability, *Caco-2* Caco-2 cells permeability, *DL* Drug-likeness

### MCAO-induced change in infarct volume and edema area

**Fig. 3:**
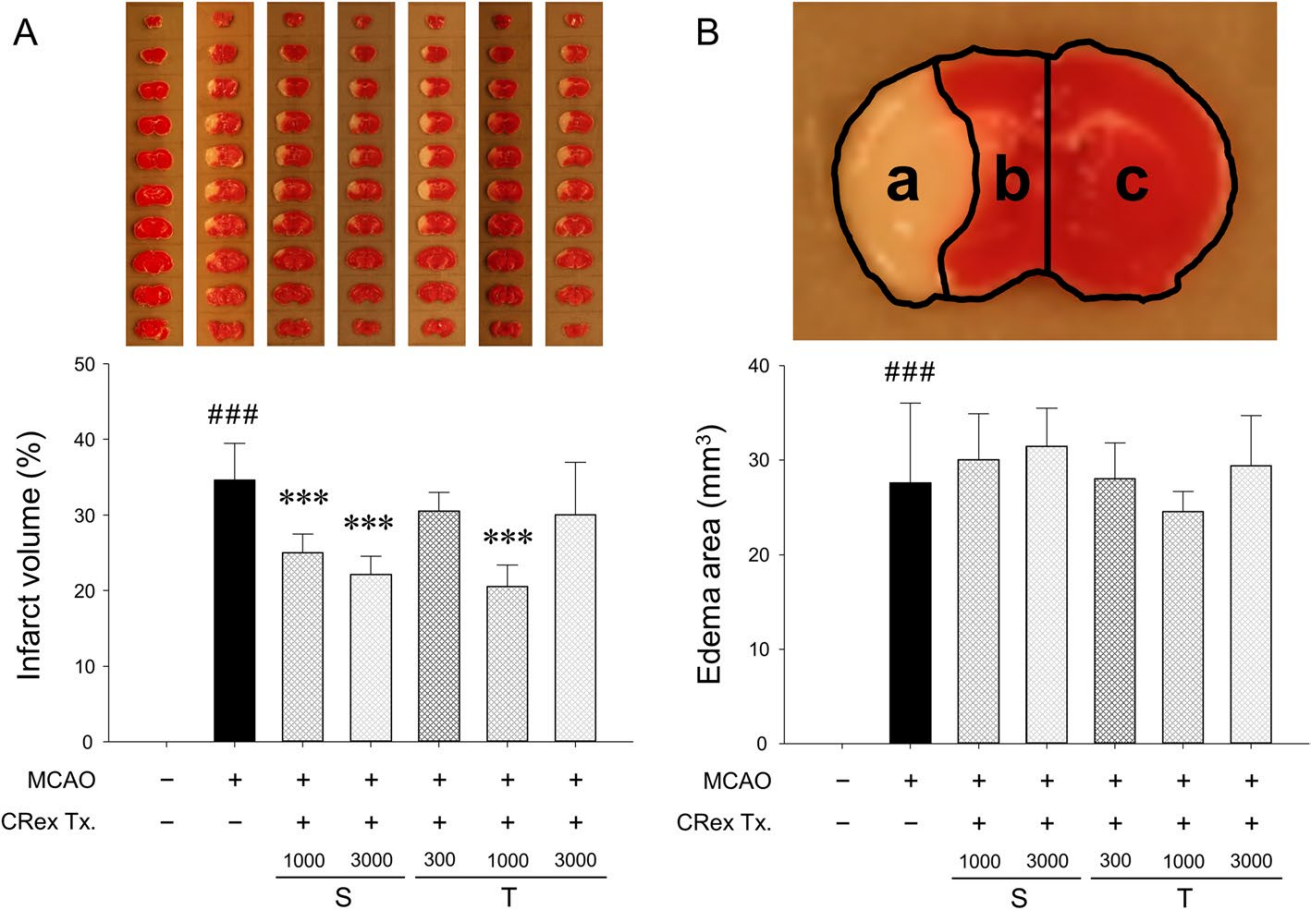
Infarct volumes and regions of edema. **A** Representative images of 1-mm brain coronal slices stained with TTC, displaying the total infarct volumes (lower column) and infarct areas 24 h after middle cerebral artery occlusion (MCAO). **B** Numerical evaluation of the entire edematous region. The results are shown as means ± SDs (*n* = 5). S, a single dose of CRex at 1,000 or 3,000 mg/kg *p.o.*; T, two doses of CRex at 300, 1,000, or 3,000 mg/kg *p.o.*; ^###^ *p* < 0.001 vs. sham-operated group; ***p < 0.001 vs. MCAO control group

Following MCAO induction, the left MCA was occluded by filament insertion for 1.5 h, cerebral infarction occurred in approximately 35% of the ipsilateral hemisphere, and when CRex samples were pretreated at a concentration of 1,000 or 3,000 mg/kg once, the extent of the cerebral infarction was significantly reduced at both concentrations. To check whether there would be activity even if the dose was reduced and two pretreatments were made; its effect was only observed at 1,000 mg/kg when pretreatment was performed at 300, 1000, and 3,000 mg/kg (Fig. 3A). Therefore, a significant effect on cerebral infarction was observed at 1,000 mg/kg; side effects might occur with increased concentration and repeated administrations. However, the CRex pretreatment did not inhibit the occurrence of cerebral edema induced by 1.5 h of MCAO (Fig. 3B). A significant difference in weight loss was observed between the sham and MCAO surgery groups but none among the mice in the MCAO surgery group (Fig. S3). No significant difference for plasma electrolyte levels was seen between the groups (Fig. S4). These results reveal that the method used for CRex administration and surgery do not affect the interpretation of the study findings.

### MCAO-induced change of NDS and forepaw grip strength

**Fig. 4:**
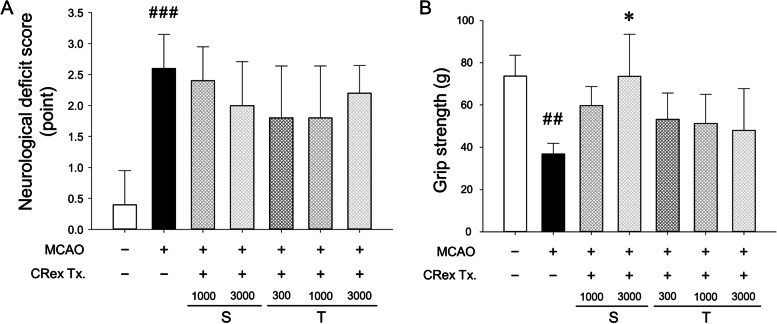
Neurological deficit (**A**) and forepaw grip strength (**B**). The results are shown as means ± SDs (*n* = 5). S, a single dose of CRex at 1,000 or 3,000 mg/kg *p.o.*; T, two doses of CRex at 300, 1,000, or 3,000 mg/kg *p.o.*; ^##^ *p* < 0.01, ^###^ *p* < 0.001 vs. sham-operated group; *p < 0.05 vs. MCAO control group. MCAO, middle cerebral artery occlusion

Cerebral infarct caused by MCAO resulted in neurological deficits in the experimental mice and decreased forepaw grip strength. CRex pretreatment did not suppress neurological deficit; however, when administered once at 3,000 mg/kg, it suppressed the decrease in the forepaw grip strength (Fig. 4A and B). These results show a slightly different tendency from those of the cerebral infarction area; this can be attributed to the greater deviation of the study results from those of the cerebral infarction area.

### MCAO-induced cognitive deficit

**Fig. 5:**
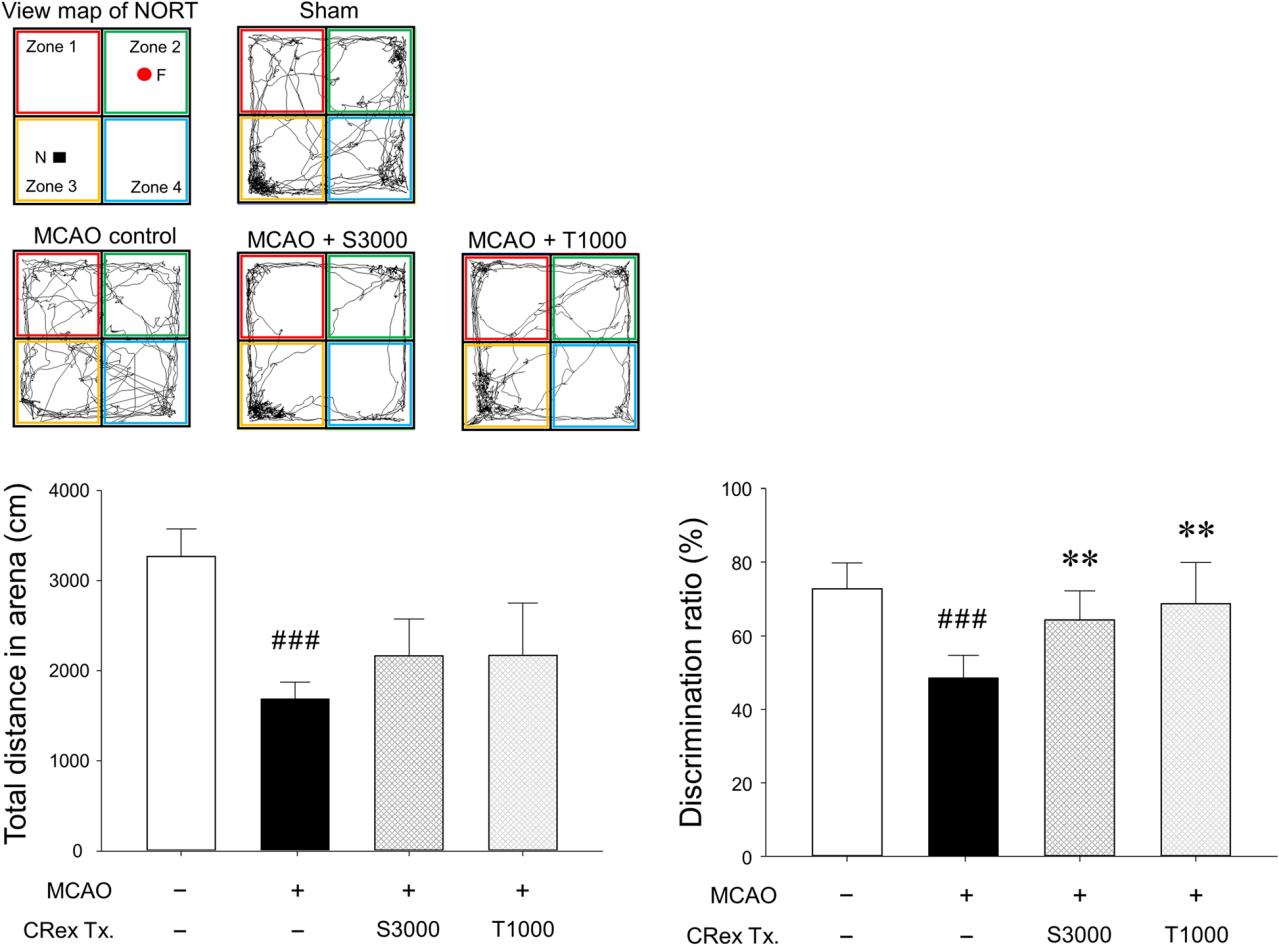
Novel object recognition test (NORT) using an open-field box. The NORT was captured with a digital camcorder at 200 lx illumination. The results are shown as means ± SDs (*n* = 5). ^###^ *p* < 0.001 vs. sham group, **p < 0.05 vs. MCAO control group. MCAO, middle cerebral artery occlusion

When MCAO-operated mice were pretreated using CRex, the most effective regimen was a single administration of 3,000 mg/kg at 1 h preMCAO, and two administrations of 1,000 mg/kg at 1 and 24 h preMCAO, which had a significant protective effect on infarct volume (Fig. 3A). The NORT was conducted to check the ability of experimental mice to recognize familiar objects under the above two conditions of CRex administration. In this study, the total distance that the MCAO control mice moved in an open-field arena for the NORT for 10 min was significantly reduced compared with the mice in the sham group; their ability to distinguish novel objects was also significantly reduced. In mice that administered the CRex, no change was observed in the total distance voluntarily moved compared to the MCAO control group; however, the cognitive function to distinguish novel objects recovered to a significant level (Fig. 5). Despite clear damage to motor function caused by cerebral infarction, the area of cerebral infarction was reduced by CRex, which resulted in less damage to cognitive function.

### Expression change in astrocytes and microglia in the penumbra area in mouse brain tissue

**Fig. 6:**
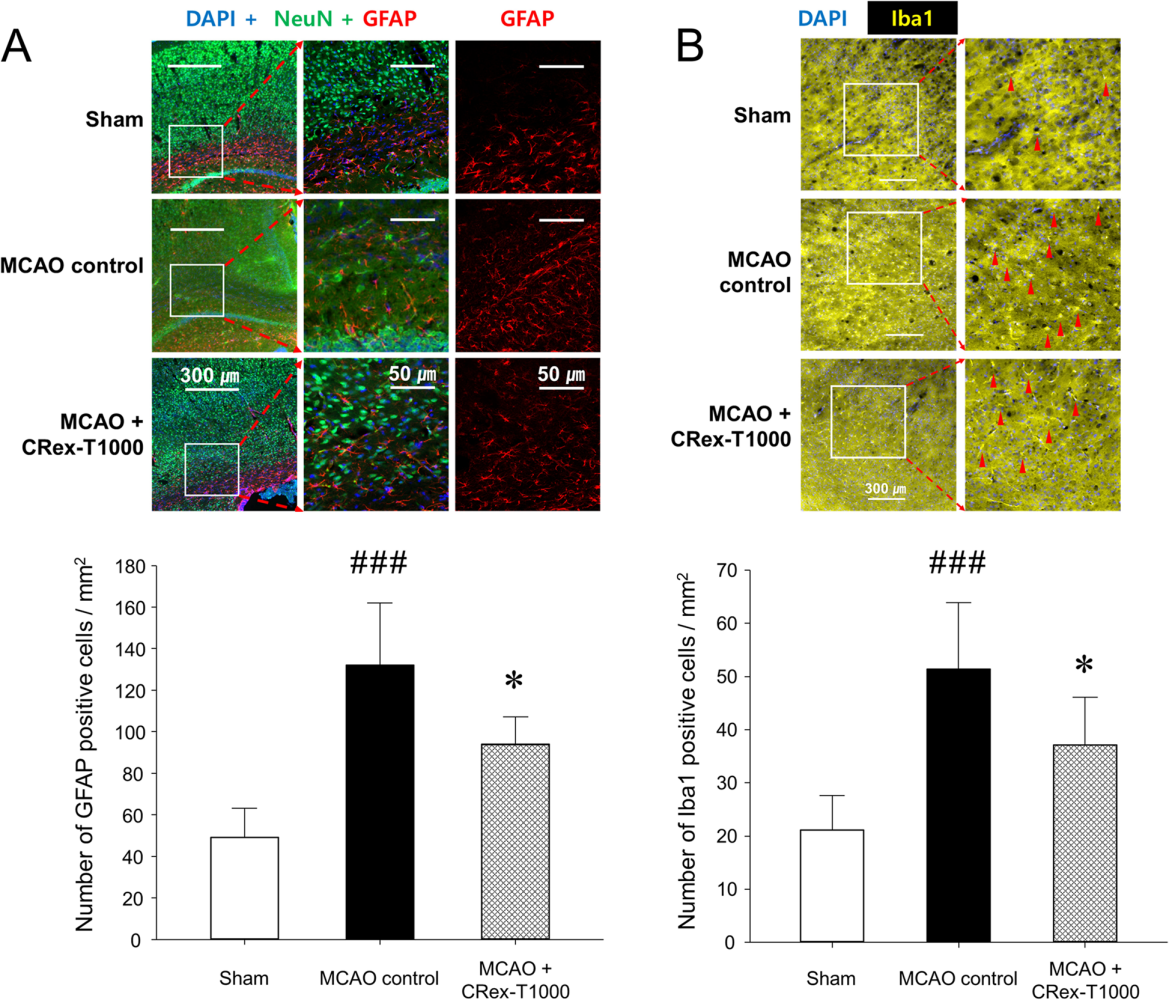
MCAO causes changes in the expression of GFAP-positive astrocytes (**A**) and Iba1-positive microglia (**B**) in the corpus callosum and cerebral cortex. The red-colored cells represent activated astrocytes (**A**), and the bright yellow-colored cells (red arrowheads) represent activated microglia (**B**). Bar graphs represent mean ± SDs (*n* = 5, per group) of IF positive cells of each antibody. ^###^ *p* < 0.001 vs. sham-operated group; *p < 0.05 vs. MCAO control group. MCAO, middle cerebral artery occlusion

Immunofluorescence staining was performed to confirm the expression of astrocytes and microglia, which are the most representative cells involved in inflammatory response in the brain. The microscopic observation area was selected as the penumbra area identified through the MCAO control group, which corresponded to the cortex and corpus callosum areas. Among the experimental groups that showed protective effects on MCAO, changes in astrocyte expression were observed in the group administered twice with 1,000 mg/kg CRex (CRex-T1000 group), which showed the most significant effects on cerebral infarction area and in the NORT. As shown in Fig. 6A, neurons are evenly distributed in the sham group; however, the degree of staining of neurons was weak around the corpus callosum in the MCAO control group. GFAP, cytoskeletal protein, is one of the main components of the glial filament of astrocytes [25, 26]. The expression of GFAP, a representative marker of astrocytes, was strongly observed in the MCAO control group. In contrast, when 1,000 mg/kg CRex was administered for 2 consecutive days before MCAO induction, GFAP expression decreased, and the staining of neurons was relatively uniform. Iba1 is a common marker for microglial activation, which characterizes its morphology and the distribution of microglia [27]. MCAO increased the expression of activated microglia in the cerebral cortex (Fig. 6B), and CRex suppressed the microglial activation.

## Discussion

CR, the rhizome of *L. chuanxiong* Hort., a perennial herbaceous plant that belongs to the *Umbelliferae* family, is widely grown in China, Korea, and Japan. Its young leaves are consumed as vegetables, and its rhizomes and roots have been reported to improve blood circulation and activate blood vessels in traditional Asian medicine [9, 10, 28]. Recent studies have shown that CR or its components exhibit various effects, such as anticancer, pain relief, anticonvulsant, anti-inflammatory, sedative, and vasodilatory effects [8, 29, 30]. Recently, Wang et al. [31] reported the microsphere-induced embolic stroke in rats and demonstrated the beneficial effects of CRex. After inducing a stroke, they administered CR ethanol extract for 14 days and explained how CR administration reduces inflammation caused by ischemia and reduces apoptosis. Several types of stroke models can be applied to rodents; however, among them, the MCAO model has the highest reproducibility [32]. In this study, we used the MCAO model to determine whether stroke symptoms could be suppressed by CRex.

Ischemic stroke affects the blood vessels that supply the brain, leading to motor and language impairment or death in severe cases, and it accounts for 87% of all stroke cases [33–36]. With the increasing prevalence of metabolic diseases and significant risk factors for stroke, stroke prevention and treatment are important areas of medical research. Therefore, research into therapeutic agents that can prevent and treat stroke is necessary [33, 35, 37].

The only treatment approved by the Food and Drug Administration for ischemic stroke is tissue plasminogen activator (tPA), which involves dissolving blood clots and improving blood flow to areas of the brain that lack blood flow. However, tPA administration is associated with increased intracranial hemorrhage (ICH) and hemorrhagic transformation. Specifically, delayed tPA administration increases the risk of edema, hemorrhagic transformation, and ICH [7, 38–42]. Therefore, drugs can be safely used for ischemic stroke with few side effects other than tPA.

Since herbal medicines such as CR contain various ingredients, network pharmacology analysis was used to check whether compounds could be bioavailable and analyze the diseases these compounds were expected to act upon. Furthermore, in various diseases caused by inflammation, it is highly likely to be active not only in vascular diseases but also in cerebral infarction. The ingredients involved in this activity include myricanone, wallichilide, perlolyrine, mandenol, and siotosterol (Fig. 2).

The recommended daily dose of CR was approximately 12 g/60 kg body weight in humans, and the corresponding dose of CRex for mice, considering the yield of CR to CRex, was 200 mg/kg; when we consider the metabolic rate of mice compared with humans [43], the corresponding dose of CRex to mice was approximately 2,000 mg/kg. Therefore, the effects of the CRex on MCAO-induced mice were observed at a concentration between 200 and 2,000 mg/kg. As a result of the preliminary study, 1,000 mg/kg showed significant effects when administered once, and similar activity was observed with 3,000 mg/kg. As a result of observing whether the effects on MCAO-induced mice repeatedly presented when mice were administered with a low concentration of CRex, no effect was noted at a concentration below 500 mg/kg, and when the concentration was increased to 3,000 mg/kg, no activity was observed. This result suggests that side effects may occur with repeated administrations of CRex at a high concentration. This study aimed to determine whether pretreatment with CRex can suppress cerebral infarction. Future studies will focus on investigating whether cerebral infarction can be similarly suppressed with posttreatment. Herbal medicines, including CR, can be administered to prevent cerebral infarction; however, active treatment of the disease requires development of herbal medicines to treat cerebral infarction outside of emergency settings. Therefore, this study aimed to confirm the minimum number of doses and determine inhibition of cerebral infarction by single or double administration.

When a cerebral infarction occurs, symptoms of paralysis such as a tingling sensation in the face, arms and legs on one side, loss of strength, and discomfort in walking appear, as well as various movement disorders [44, 45]. Although to a lesser extent than in humans, motor impairment also occurs in rodents [46]. In this study, NDS changes and forepaw grip strength were evaluated to determine the degree of MCAO-induced motor function abnormalities. Consequently, significant motor functional deficits were observed in the MCAO control group; however, NDS showed no significant change following the administration of the CRex at various concentrations (Fig. 4A), and in the forepaw grip strength, a significant decrease in grip strength was suppressed only in the experimental group administered 3,000 mg/kg CRex 1 h before MCAO induction (Fig. 4B).

Stroke often manifests cognitive function impairment along with physical dysfunction, sleep disturbance, behavioral and personality changes, and neuropsychological changes. It can affect memory, thinking ability, planning ability, language function, attention, and daily living skills [47, 48]. MCAO-induced ischemic stroke in mice showed a significant reduction in spontaneous motor activities and DR in the NORT, and CRex suppressed such deficit in the DR (Fig. 5).

The expression of cells involved in the inflammatory response in brain tissue was evaluated using IF staining. MCAO induced an increase in GFAP expression in the cerebral cortex and corpus callosum, whereas CRex inhibited such activation (Fig. 6A). Furthermore, CRex inhibited the MCAO-induced Iba1 activation (Fig. 6B), suggesting that CRex has anti-inflammatory properties. Because microglia and astrocytes play major roles in controlling the inflammatory response in the central nervous system, their activation causes the release of cytokines such as tumor necrosis factor-α, interleukin (IL)-1β, and IL-6, which in turn trigger an inflammatory response [44–48]. The above results confirmed that MCAO may cause an inflammatory response, which may lead to further brain damage, and that CRex can reduce damage by regulating the activity of cells involved in the inflammatory response in the brain.

This study confirmed that pretreatment with CRex inhibits cerebral infarction in mice. However, since the onset of cerebral infarction cannot be predicted in humans, determining the posttreatment efficacy with CRex would be more significant. In addition, one-time high-dose CRex administration suppressed cerebral infarction; however, administering the same concentration twice tended to increase the cerebral infarction, confirming that the concentration and number of doses significantly influence the therapeutic effect. Although this study is limited to confirming the effect of pretreatment on suppressing cerebral infarction, a better-structured follow-up study can be conducted based on the current results. In our follow-up study, we aim to confirm the efficacy of posttreatment with CRex against cerebral infarction by studying the main components of CRex that play key roles in inhibiting cerebral infarction.

## Conclusions

In this study, to evaluate the neuroprotective effects of CR on ischemic stroke, mice with MCAO-induced ischemic stroke were administered CRex, a methanol extract of CR. To induce an ischemic stroke, the MCA was blocked for 1.5 h, and various biological markers of stroke were assessed. Pretreatment with CRex substantially decreased the infarct volume. CRex also inhibited a significant reduction in recognition function. Ultimately, CRex lowered inflammatory response through the control of astrocyte and microglia activation.

### Supplementary Information

**Supplementary Material 1.**

## Data Availability

All data in this study are available from the corresponding author on reasonable request.

## Abbreviations

ADME: Absorption, distribution, metabolism, and excretion
CR: Chuanxiong Rhizoma
CRex: Chuanxiong Rhizoma methanol extract
CVDs: Cerebrovascular diseases
DR: Discrimination ratio
GFAP: Glial fibrillary acidic protein
Iba1: Ionized calcium-binding adaptor molecule 1
IF: Immunofluorescent
NeuN: Neuronal nuclear
NORT: Novel object recognition test
MCAO: Middle cerebral artery occlusion
rCBF: relative cerebral blood flow
TCMSP: Traditional Chinese Medicine Systems Pharmacology Database and Analysis Platform
