# Supplementary material for: Neuroprotective effects of methanolic extract from Chuanxiong Rhizoma in mice with middle cerebral artery occlusion-induced ischemic stroke: suppression of astrocyte- and microglia-related inflammatory response
Source: BMC Complement Med Ther. 2024 Apr 4;24:140. doi: 10.1186/s12906-024-04454-w (PMC10993527; doi:10.1186/s12906-024-04454-w)
Supplement: Supplementary file 1 — Supplementary Material 1. [file 12906_2024_4454_MOESM1_ESM.docx]

**Supplementary Figures**

**
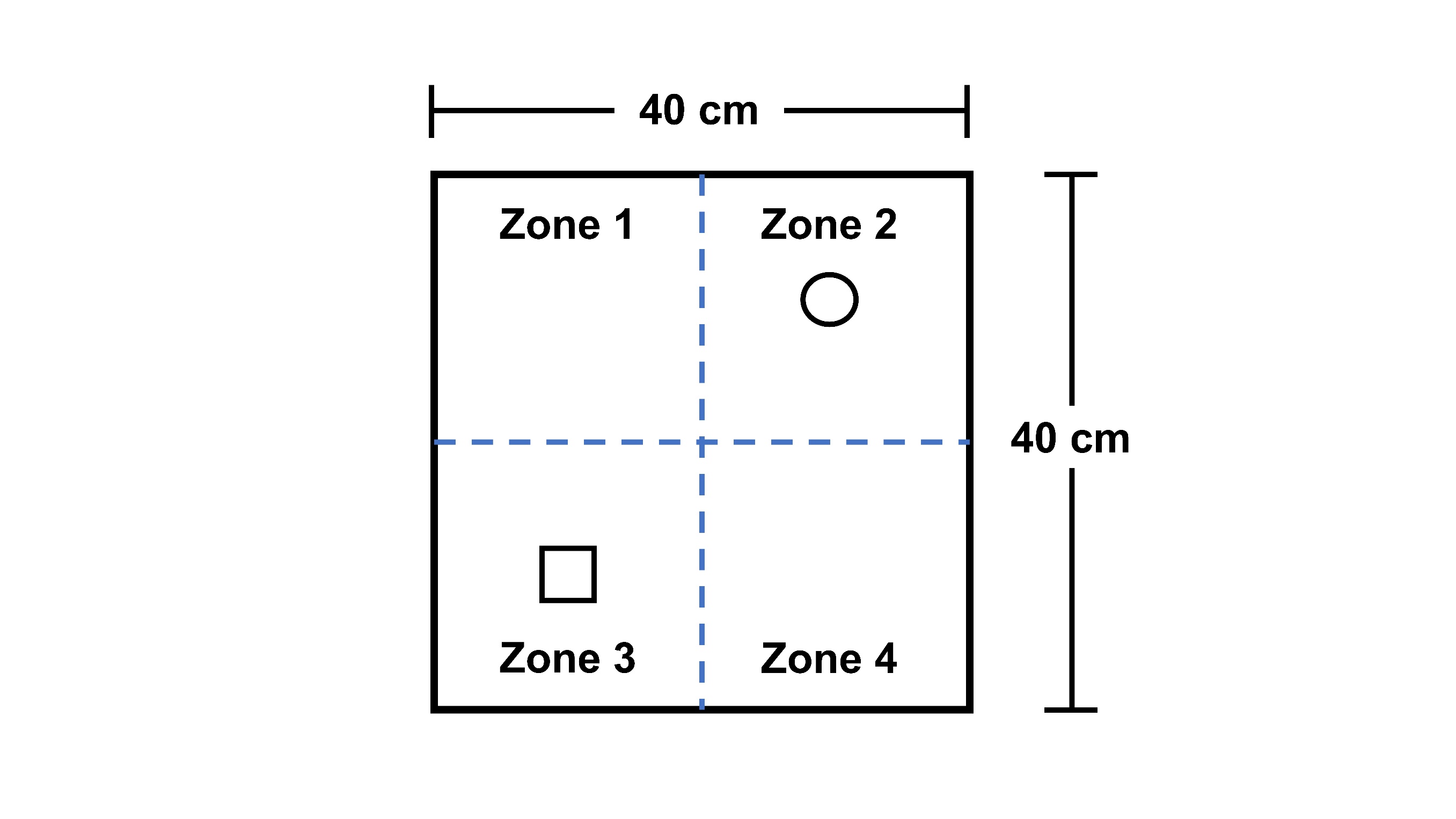
Fig. S1** Specifications of the open field box for novel object recognition test (NORT) measurement. The objects in zones 2 and 3 were secured using double-sided tape to prevent them from moving easily.


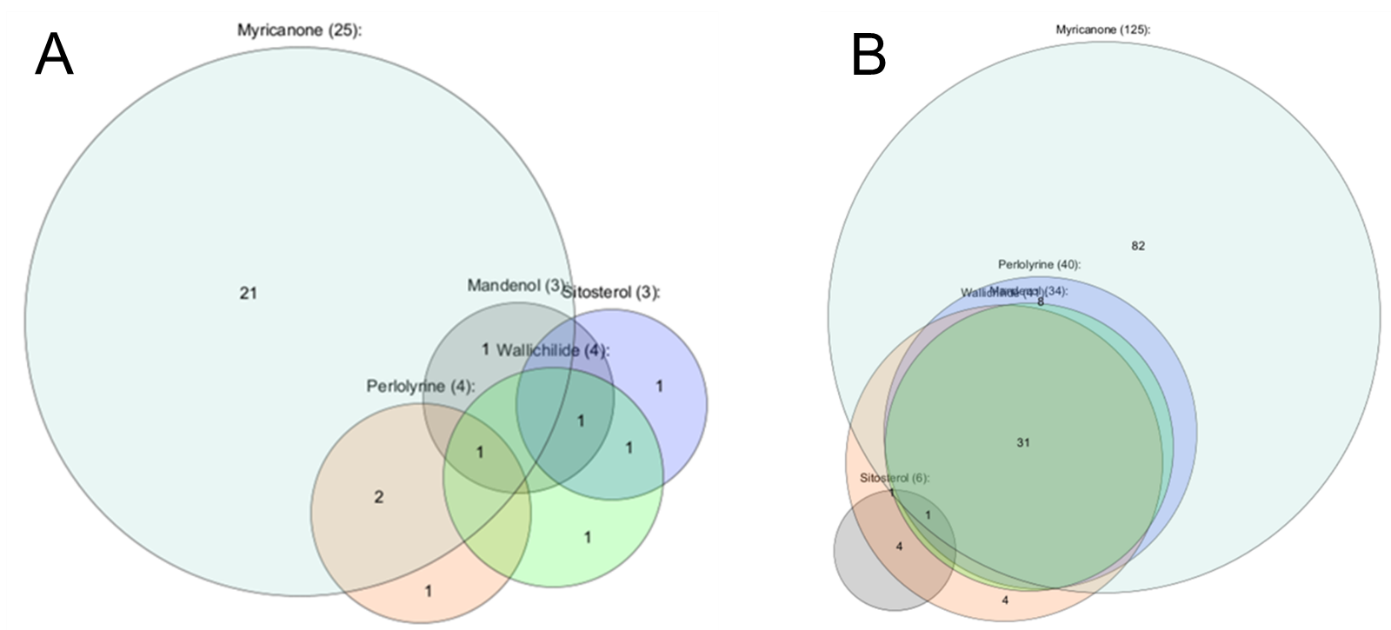


**Fig. S2** Euler diagram expressing the common target proteins and disease networks shared by the components of Chuanxiong Rhizoma extract.


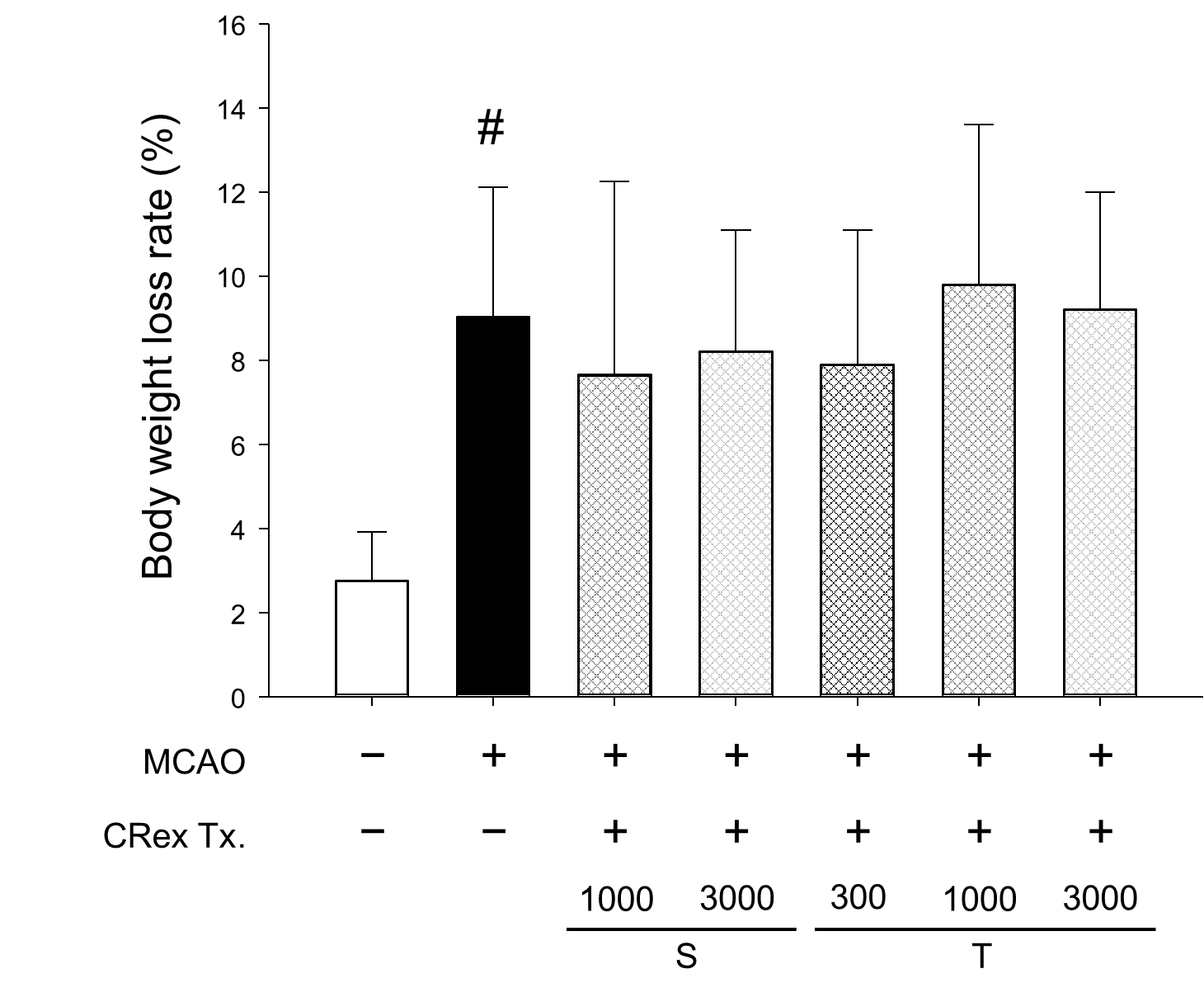


**Fig. S3** Rate of body weight loss before and 24 h after middle cerebral artery occlusion (MCAO) surgery. The MCAO control group showed significant body weight loss compared to the sham surgery group. The results are shown as means ± SDs (n = 5). S, a single dose of CRex at 1,000 or 3,000 mg/kg *p.o.*; T, two doses of CRex at 300, 1,000, or 3,000 mg/kg *p.o.*; ^#^*p* < 0.05 vs. sham-operated group.


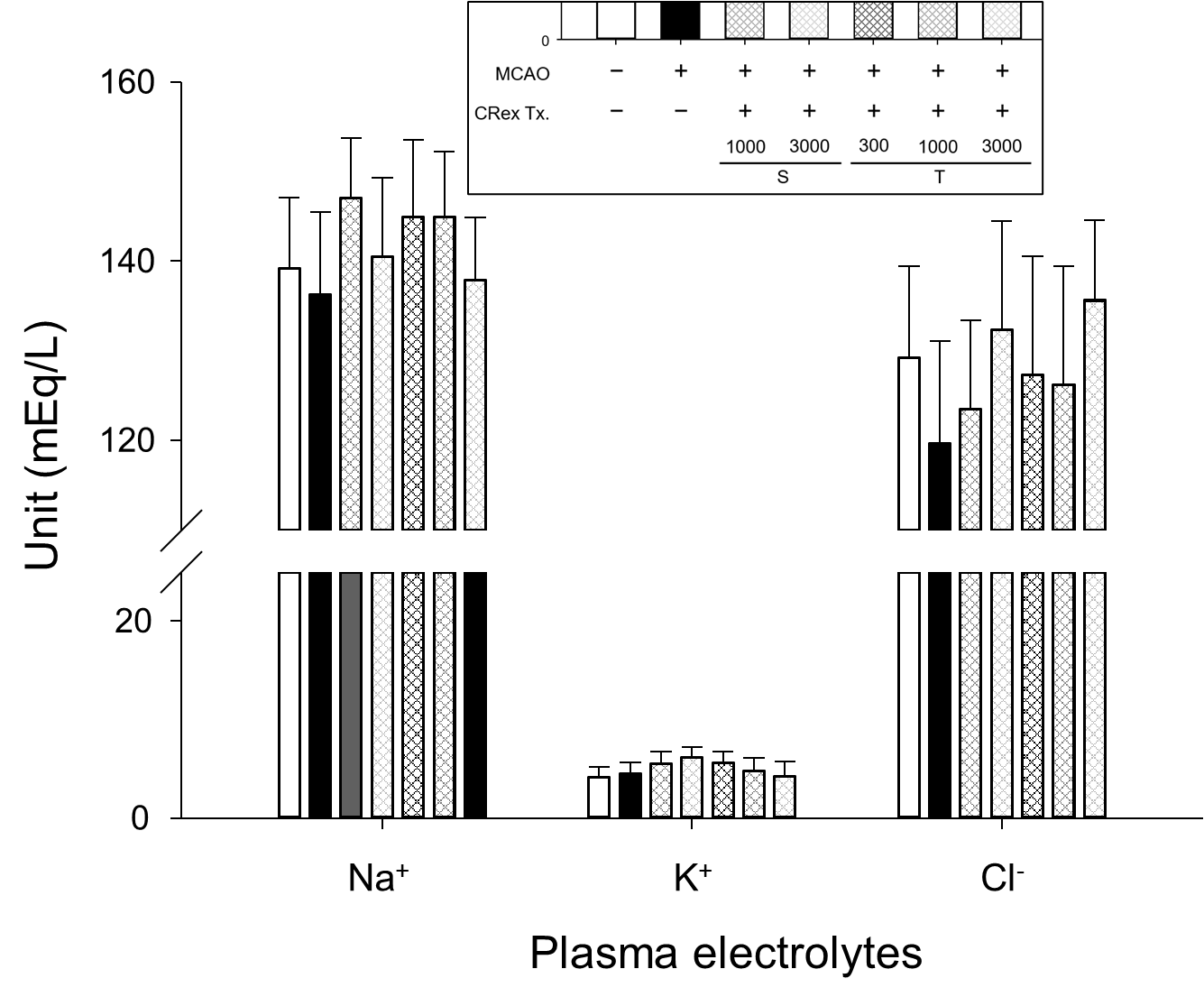


**Fig. S4** Influence of middle cerebral artery occlusion (MCAO) surgery in mice and the effect of CRex administration on plasma electrolytes. Plasma samples were obtained by collecting whole blood at the end of the experiment. After plasma samples were taken, the concentrations of Na^+^, K^+^, and Cl^−^ were measured. The results are shown as means ± SDs (n = 5). S, a single dose of CRex at 1,000 or 3,000 mg/kg *p.o.*; T, two doses of CRex at 300, 1,000, or 3,000 mg/kg *p.o.*
